# New Zealand deaf adults’ perspectives on speech-language therapy for deaf children

**DOI:** 10.1093/jdsade/enag011

**Published:** 2026-04-11

**Authors:** Jayne Newbury, Rosie Lamb, Toby Macrae

**Affiliations:** School of Psychology, Speech and Hearing, University of Canterbury, Christchurch 8140, New Zealand; School of Psychology, Speech and Hearing, University of Canterbury, Christchurch 8140, New Zealand; School of Psychology, Speech and Hearing, University of Canterbury, Christchurch 8140, New Zealand

## Abstract

Speech-language therapy (SLT) within deaf education in Aotearoa New Zealand remains influenced by oralist traditions. Māori deaf individuals also face barriers to their culture and native language. This qualitative study explored deaf adults’ experiences and perceptions of SLT, including how SLTs should best support deaf children. Focus groups and interviews were conducted with 11 deaf adults (aged 28–65) who had engaged with SLT. Data were generated in New Zealand Sign Language or English and analysed using reflexive thematic analysis. *Ensuring deaf children thrive and flourish* was the overarching theme, with three subthemes (1) *Hearing differences create a unique experience* (2) *Consider the child’s unique ecosystem* and (3) *The profession’s responsibilities*. Participants wanted culturally affirming collaborative SLT practice, including signed and spoken languages. They prioritised early language access, communication success, strong therapeutic relationships and diversifying the workforce to ensure deaf children flourish.

Speech-language therapists (SLTs) are key professionals who can support deaf children and their parents to make decisions around communication options. However, deafness is not common with only two to three in every 1,000 children being identified with moderate to profound permanent hearing differences by age 19 years (Ministry of Health, 2020). Deaf children are a heterogeneous group, varying in hearing levels, interventions and linguistic access, and these differences help shape their wide-ranging outcomes. Over 90% of deaf children are born to hearing families (Mitchell & Karchmer, 2004). Such families heavily rely on professional advice for how to best support their deaf child’s communication and are unlikely to have met deaf people previously or to know a signed language (Terry & Rance, 2023). However, the SLTs themselves may not be well informed on deaf adults’ perspectives on how a deaf child’s communication can be best supported with limitations in both training and opportunities to develop specialist expertise (Blaiser & Mahshie, 2022; Jones & Roberts, 2024). This article reports on deaf adults’ perceptions of SLT that they experienced as children or observed as a professional or parent of a deaf child, with a view to informing service developments.

This article uses “deaf” to represent all individuals with moderate or more severe hearing differences, including developmental and acquired deafness, and “Deaf” for those identifying with Deaf culture. Deaf culture views deafness as a source of pride rather than a disability and is grounded in signed languages for daily communication (Deaf Aotearoa, 2024).

## Historical and current context of deaf education in New Zealand

Historically, deaf education in New Zealand, as in many Western countries, was shaped by oralist traditions that prioritised speech and lip-reading while excluding sign languages (McKee, 2017). These approaches reflected ableist assumptions that framed hearing and speaking as superior (Bauman, 2004; Eckert & Rowley, 2013; Wolbring, 2008), in some cases leading to language deprivation and long-term negative impacts for deaf children (Cheng et al., 2019; Gulati, 2018; Hall et al., 2019). Although universal newborn hearing screening and technology, such as cochlear implants have improved sound access, devices do not replicate natural hearing (Lesica, 2018). Full language access through sign has been declared a right of deaf children (World Federation of the Deaf, 2016).

For Māori deaf (tangata tūrī; indigenous people of New Zealand), these challenges are compounded by the enduring effects of colonisation and breaches of te Tiriti o Waitangi (the Treaty of Waitangi), which promised Māori self-determination but was largely ignored in practice (Ministry of Justice, 2016; Walker, 2016). Assimilationist education marginalised the Māori language and Māori cultural values, leaving Māori deaf with limited access to both Deaf and Māori identities (Crisp, 2010; Smiler & McKee, 2006; Wilson & Pointon, 2019). Māori deaf people therefore experience compounded colonisation, racism, and ableism.

## Theoretical perspectives

Different theoretical frameworks offer contrasting ways of understanding deafness within Western literature. The medical model conceptualises deafness as an individual impairment to be “cured” or “normalised” to meet dominant societal norms (Haegele & Hodge, 2016). In contrast, Deaf culture embodies a sociolinguistic and cultural model, framing deafness not as a deficit, but as a natural variation grounded in shared language, identity, and community (Ladd & Lane, 2013). Building on ecological perspectives, Cue (2020) proposed the Deaf Ecological Systems Model, which highlights how medicalised attitudes, inaccessible education systems, and family contexts interact to influence a deaf child’s linguistic, cognitive, and social development. This model underscores the importance of viewing deafness within the child’s broader ecosystem, reminding professionals to design assessment and intervention practices that are holistic, relational, and contextually responsive. The current study uses the sociolinguistic, cultural and ecological models of Deafness as its framing (Cue, 2020; Ladd & Lane, 2013).

## Language development and supports for deaf children in NZ

In New Zealand, deaf children are supported through public health (audiology), education funding (language and learning), not-for-profit services (cochlear implant habilitation) and a range of private services. A Universal Newborn Hearing Screening and Early Intervention Programme (UNHSEIP) was rolled out nationwide by 2010 to identify deaf children shortly after birth (Ministry of Health, 2014). Identified babies are referred for publicly funded audiology, including hearing aids or cochlear implants depending on needs, eligibility, and family preferences. Note language supports are not limited to those provided by SLTs. Families are offered an Advisor on Deaf Children, from the Ministry of Education Learning Support service. A newer initiative, First Signs (see https://firstsigns.co.nz/) provides tutors to teach families New Zealand Sign Language (NZSL) and connects them to the Deaf community, but resourcing remains low (Ministry of Education & Fitzgerald & Associates, 2019). The Ministry of Education Learning Support service provides application-based funding for deaf children to access teaching assistants, NZSL interpreters and NZSL tutors to support their communication in mainstream classrooms (Ministry of Education, 2022).

There are no published reports on the scope of SLT services to deaf children from New Zealand or the languages their services are provided in. However, in a broader national survey of multilingual practice of 113 paediatric SLTs, fewer than 10% of SLTs reported functional or better competence in Māori and less than 1% for NZSL, while it is assumed all were proficient in English (Newbury et al., 2020). Given Māori was the most common additional language of SLTs, it can be assumed SLTs services in NZ are provided in English as a default, with a few exceptions.

Most deaf children are mainstreamed under a policy of inclusion (Powell & Hyde, 2014). As an unintended consequence, this may limit daily contact with other deaf peers or adults. Deaf children in mainstream education who require communication supports can be referred to Ministry of Education Learning Support Service SLTs. Their practice model, He Pikorua, is a collaborative child- and family- centred service, focused on inclusion through ecological, culturally affirming, strengths-based, evidence informed supports (Ministry of Education, 2021; see also https://hepikorua.education.govt.nz/). These therapists have limited opportunities for specialisation, working with children with communication difficulties across aetiologies aged 0–21 years. Demand for Learning Support services exceeds capacity, including for SLT services (Stanford, 2025). Children with cochlear implants, however, receive regular individualised SLT services through private not-profit specialist centres (see https://audiology.org.nz/for-the-public/about-hearing-loss/cochlear-implants/). These therapists specialise in services for deaf children with cochlear implants. Their practice model is not publicly available; however the emphasis is on auditory verbal habilitation.

Ko Taku Reo Deaf Education New Zealand, the national provider of deaf education, operates two preschools, satellite classrooms, and itinerant services with professionals including SLTs, resource teachers, interpreters, and teaching assistants (see https://www.kotakureo.school.nz/parents-and-whanau). Although Ko Taku Reo promotes a trilingual/tricultural approach (English, NZSL and Māori), this appears aspirational in terms of teaching practice (Ko Taku Reo Deaf Education NZ, 2025). An Education Review Office evaluation (Education Review Office, 2024) identified concerns with student achievement, attendance, governance, and leadership. Recommendations included staff development in NZSL as a medium of instruction, improved assessment and teaching practices, and greater understanding of Deaf culture. See Powell and Hyde (2014) for further history of deaf education in New Zealand.

### SLT in New Zealand, ethics and scope of practice

The New Zealand Speech-language Therapists’ Association (NZSTA) Code of Ethics (NZSTA, 2020) provides a values-based framework for SLTs working across employers in New Zealand. It requires therapists to enhance client and family wellbeing and avoid causing harm. Practitioners must work within their competence and provide evidence-based and culturally responsive practice. They must explain information appropriately, including findings, limitations and interpretations of services provided. Integrity is upheld through honouring Te Tiriti o Waitangi and being honest and respectful to clients, being mindful of their rights and sensibilities. Fairness requires non-discrimination, equitable resource allocation, communication of consumer rights, and prompt conflict management.

The NZSTA Scope of Practice (NZSTA, 2012) defined the activities, knowledge, skills, and ethical behaviour expected of SLTs, while noting practice must remain within each individual’s competence. It emphasised continuous revision to reflect emerging practice areas and cultural sensitivity under Te Tiriti o Waitangi, urging therapists to consider diverse worldviews, languages, and socioeconomic factors. It formally endorsed the International Classification of Functioning Disability and Health (ICF) framework (World Health Organisation [WHO], 2001), shifting focus from cause to impact and focusing on holistic client-centred services. Services included assessment, goal setting, treatment, counselling, family support, capacity building, and advocacy for minority languages. Rationale for service included diagnosis, improved communication, wellbeing, and participation across contexts. Models of service provision included naturalistic and clinic-based intervention, family- and client-centred approaches (Allen & Petr, 1996) and holistic care. This document has recently been superseded (NZSTA, 2025), however the earlier version is most relevant to the data presented in this study. The recent update does not specify models of practice.

### SLT practices internationally, ableism and audism

SLT emerged in the early 1900s around “correcting” speech (Duchan, 2002), reflecting ableist paradigms privileging one “ideal” mode of communication (St. Pierre & St. Pierre, 2018). Anti-ableist efforts now include stuttering-affirming practices (Sisskin, 2023) and neurodiversity-affirming frameworks (Gaddy & Crow, 2023), which value diverse communication modes and promote identity affirmation. However, more effort could be made to align SLT services with Deaf culture. While NZSTA’s scope of practice (2012) and code of ethics (2020) require culturally responsive practice, therefore NZSL competence for those working with Deaf people, recall that fewer than 1% of SLTs reported functional competence in NZSL (Newbury et al., 2020).

Auditory Verbal Therapy (AVT) is a prominent approach for deaf children, focusing solely on developing spoken language through listening (Auditory Verbal UK, 2023). Methods include restricting lip-reading cues and using tactile feedback for voicing. This is an ableist approach from a Deaf perspective as AVT prioritises speech and auditory functioning as markers of success, and does not include supports for signing, Deaf identity, or environmental accommodations. However, family-centred care supports the right of parents to make choices for their deaf children (Allen & Petr, 1996). Research shows AVT can support deaf children to reach spoken language levels comparable to hearing peers, but systematic reviews highlight limited quality evidence and a lack of randomised trials (Binos et al., 2021; Noel et al., 2023). AVT risks language deprivation, if parents opt for an oral-only approach. Other evidence suggests sign language may reinforce spoken language for children with cochlear implants and therefore there may be an advantage to giving deaf children access to both sign and spoken languages early on (Delcenserie et al., 2024; Klaudia, 2013).

The ICF model (WHO, 2001) prompts SLTs to consider the impact of the environment and personal factors on a client’s participation and activities; a broader scope than oralist methods, which focus on reducing the “impairment.” Similarly a recent systematic review of deaf children’s experiences of hearing in everyday life suggested individualised changes in the social and physical environment around deaf children could improve their day-to-day participation, inclusion and communication (Nightingale et al., 2025), for example, training communication partners to use strategies to manage the experience of hearing, such as reducing their rate of speech, avoiding overlapping speech and making sure their mouth is visible, along with providing quiet listening environments.

International research is still dominated by professional perspectives, rather than those of deaf individuals. Some studies from experts in the U.S. highlight cultural gaps between SLTs and deaf communities, urging respect for Deaf identity, use of sign language, the need for high expectations, and functional goals (Cripps et al., 2016; Secora et al., 2025; Veyvoda et al., 2019). It should be noted that Secora et al.’s expert panel, the most recent study, was made up of 53% deaf participants, representing an important shift towards respecting deaf experiences in framing services. Parents’ views are better represented than deaf children’s in the literature (Kelly & Drasgow, 2020). There are two studies involving deaf participants’ views on SLT to our knowledge, and only one of these is published (Greene et al., 2023; Sankey, 2019). These recommend combined signed/spoken approaches, bicultural practice, support for identity, and avoidance of deficit assumptions. These findings align with the NZSTA’s scope of practice (2012), which requires culturally responsive practices and frames wellbeing and quality of life as outcomes.

Therapeutic relationships are crucial. Reviews by Bright and Reeves (2022) and Hansen et al. (2024) showed client engagement depends on trust, validation, and attunement to communication needs. Participants in these studies reported “reading” their clinicians, stronger relationships when communication was adjusted and personhood affirmed, and disengagement when needs were ignored. There is little research on how relationships are built between SLTs and deaf children. However, Sankey (2019) in their master’s thesis, found acceptance or use of sign language enhanced comfort and rapport for Deaf adults.

## Summary

In summary, despite a sound code of ethics and scope of practice (NZSTA, 2012, 2020), some SLT services for deaf children in New Zealand may continue to reflect historical oralist paradigms, ableism, and limited recognition of deaf adults’ perspectives (St. Pierre & St. Pierre, 2018). In addition to this, deaf Māori also experience a lack of support for Māori culture and access to the Māori language (Crisp, 2010; Smiler & McKee, 2006; Wilson & Pointon, 2019). Although full access to language through sign has been declared a right of deaf children (World Federation of the Deaf, 2016) there remains a critical knowledge gap concerning how deaf people in New Zealand, in all their cultural and linguistic diversity, envision and evaluate SLT support, including the therapeutic relationship. To address this gap, the present study explored deaf individuals’ perspectives on SLT within the local social and cultural context of Aotearoa New Zealand. Guided by sociolinguistic, cultural and ecological models of deafness (Cue, 2020; Ladd & Lane, 2013), we position deaf adults as expert informants and centre their perceptions of SLT services within a wider ecological context. In doing so, we aimed to generate practice-relevant insights to inform more responsive and equitable service provision.

Specifically, we asked:

1. What are deaf people’s experiences of SLT?
2. What are deaf people’s perspectives on SLT?
3. How do deaf people think SLTs should support deaf children?

## Methods

The authors are three New Zealand speech-language therapists whose professional and personal experiences shape this research. The first author (Newbury) has teaching, clinical, and research experience in child communication, and supervises postgraduate work in deaf studies. The second author (Lamb) has extensive experience in deaf education, private practice with deaf children, and clinical education. As a fluent user of New Zealand Sign Language (NZSL), she is well connected with Deaf communities. This research was prompted by her conversations with Deaf people, where distrust and dissatisfaction with SLT were expressed. The third author (Macrae) has clinical and research expertise in speech sound development in children.

As hearing professionals working within predominantly hearing institutions, we recognise the power and privilege we hold in shaping how deafness is taught, understood, and supported in speech-language therapy. Given the tension between the medical model and the sociolinguistic, cultural and ecological models of deafness, our shared motivation is to learn from the perspectives and experiences of deaf adults and to reflect critically on our own assumptions, language choices, and professional practices. We acknowledge that our positionalities as hearing SLTs may both enable and limit our interpretations. We approached the research with an explicit commitment to learning from and being accountable to deaf participants.

This study was situated within a transformative research paradigm (Mertens, 2008), with a commitment to social justice and the critique of existing power structures. Within this paradigm, ethical considerations (axiology) extend beyond procedural ethics to include an active engagement with participants’ communities, their current challenges, and the structural conditions that maintain the status quo. Ontologically, the study assumes that realities are socially constructed and shaped by individuals’ social positioning and access to power, which in turn influence whose perspectives are included in defining what counts as “truth.” Epistemologically, knowledge is viewed as situated within specific social and historical contexts, and as co-constructed through reciprocal relationships between researcher and participants. These assumptions informed the design, conduct, and interpretation of the research.

The principles of Kaupapa Māori Research (Smith, 2015) and the Deaf Accessible, Cultural and Ethical Studies (AcCES) Checklist (Kenway & Riley, 2021) were used to design the methods, while working in the transformative research paradigm (Mertens, 2008). Therefore, initial consultations with stake holders in the d/Deaf and Māori communities were undertaken to check the study was valued by these communities before the research went ahead. Changes were made to the proposed semi-structured interview questions to improve clarity in response to community feedback. A formal consultation process with the Ngāi Tahu Consultation and Engagement Group was also successful. An application to the Human Research Ethics Committee at the University of Canterbury was approved (HREC 2022/149).

## Recruitment

The second author invited those in her professional networks who had expressed interest in the project during planning phase discussions. People who agreed to be participants were asked to pass on the information to additional potential participants. Social media was also used to share recruitment materials via organisations and community groups within the context of the deaf community, for example, Deaf Clubs around Aotearoa, Deaf Māori groups, cochlear implant programs, Deaf Aotearoa and the National Foundation for Deaf and Hard of Hearing. Recruitment was undertaken in 2023. Unsurprisingly, given the low incidence of childhood deafness, the small population of New Zealand, and multiple demands on deaf adults’ time, recruitment was challenging.

Multiple methods of contact were offered to potential participants, namely, email, phone/video and via social media. A custom online contact form was developed, which included videos in NZSL. This form aimed to reduce the amount of written English that the person was required to read and produce, in comparison to a standard written message, for example, email. These measures were taken to ensure the study was accessible to Deaf participants (Kenway & Riley, 2021). Following an initial expression of interest in the project, the information sheet and consent form was sent to the potential participant. The information sheet and recruitment NZSL videos were recorded as a dialogue between the second author and a culturally Deaf person who is active in the Deaf community, format with to align with cultural norms in the Deaf community (Kenway & Riley, 2021; Mckee et al., 2013; New Zealand Sign Language Board, 2023). The information sheet included information about the hearing status, motivation and profession of the second author, and how potential participants could connect with her in NZSL or English. It included encouragement for participants to honestly share their experiences and perspectives about SLT and acknowledged this may be difficult given her profession. All participants had an initial meeting with the second author pre data generation to build trust, discuss the project and answer questions. This is an important step to support agency and ethical practice in Deaf research, and also aligns with Māori culture (Kenway & Riley, 2021; Smith, 2015).

## Participants

The participants in this study had to be adults over the age of 18 years, who had hearing differences that were identified in childhood and who currently lived in New Zealand. Sixteen people made initial contact with the researcher, five people did not return contact, therefore did not become participants. Eleven signed consent forms and were included in the study. Eleven was a sufficient sample size, as data gathered from this group were rich and coherent. Data saturation and reliability are not aims in reflective thematic analysis (Braun & Clarke, 2019). Five participants were aged 28–33 years and six were aged 48–65 years. Four of the participants lived in the North Island and seven in the South Island. Specific ages and places of residence will not be reported as it may compromise their anonymity, due to the NZ deaf community being small (Singleton et al., 2014). Participants chose their own pseudonym. All but one participant received SLT as a child in New Zealand, and many later engaged with therapists, either as colleagues or as parents. Data on hearing status and device use was not systematically collected. See Table 1 which presents participant characteristics.

**Table 1:** Participant characteristics.

Pseudonym	Age bracket (years)	Ethnicity	Experiences with SLT	Languages used
Will	28–33	New Zealand European	As a child	Spoken and written English, NZSL
John	28–33	New Zealand European	As a child	Spoken and written English, some NZSL
Ngaroma	28–33	Māori	As a colleague	Māori, spoken and written English, some NZSL
Mary	48–65	New Zealand European	As a child As a parent of a deaf child	NZSL, written English
Rachel	48–65	European	As a child As a parent of a hearing child	British Sign Language, NZSL, written English
Allen	48–65	New Zealand European	As a child As a parent of a hearing child	NZSL, spoken and written English
James	48–65	European	As a child As a colleague	NZSL, written English
Aseefa	28–33	Indian	As a child As a colleague	NZSL, written English
Hannah	28–33	New Zealand European	As a child As a colleague As a parent of a deaf child	NZSL, written English
Chloe	48–65	New Zealand European	As a child As a colleague As a parent of a hearing child	NZSL, spoken and written English
Evelyn	48–65	European	As a child As a colleague	Spoken and written English

Note. NZSL: New Zealand Sign Language; SLT: Speech and language therapy*.*

## Data generation

Data was generated with participants using focus groups (two or more people) and semi-structured interviews (one person). Focus groups and interviews took place via Zoom (Zoom Video Communications, 2023). Audio (when spoken language was used) and video were recorded for transcription. While the initial plan was for focus groups only, several participants indicated they did not feel comfortable speaking on this topic in a group and requested an individual interview. Therefore, in alignment with the principles of participatory research, we offered all participants the choice between a focus group or interview (Kenway & Riley, 2021). They also chose the language used (NZSL or spoken English). The second author conducted all focus groups and interviews and has advanced proficiency in NZSL. Focus-group members were split into pre-1990 (34 years and over) and post-1990 (33 years and under) birth cohorts. Those born post-1990 may have accessed cochlear implant technology (The Hearing House, n.d.) as well as benefited from evolving educational approaches for deaf children. Distribution of participants, language used and data generation methods is displayed in Table 2. All focus group meetings and interviews began with introductions to build rapport, an explanation of the study and the motivation behind it, and a chance to connect on a personal level first, as aligns with Deaf and Māori cultures (Kenway & Riley, 2021; Smith, 2015). The duration ranged from 30–75 min.

**Table 2:** Data generation methods.

Data generation method	Participants	Language used
Focus group 1	Will and John	Spoken English
Focus group 2	Mary, Rachel, Allen and James	NZSL
Focus group 3	Aseefa and Hannah	NZSL
Interview 1	Ngaroma	Spoken English
Interview 2	Chloe	NZSL
Interview 3	Evelyn	Spoken English

Note. NZSL: New Zealand Sign Language.

The interviews and focus groups consisted of discussion of three broad questions which were aligned with the initial research questions:

1. What is your experience of SLT?
2. What is your perspective on the SLT profession?
3. Do you think that SLT supports deaf people well? If not, what could be different?

These questions were elaborated on and re-phrased, to support understanding as required. The second author was proactive in requesting and providing clarifications to ensure clear communication. Question three required further exploration in all data generation sessions. For example, the following additional questions were asked when a communication breakdown was noticed: What do you think SLTs need to know when working with deaf people? How could SLTs support deaf children’s communication skills? When you think about SLTs, as a group, or as a profession, what comes to mind?

Notes were taken by the second author, directly after the conclusion of the focus group or interview. These notes were reflexive and often included the most salient aspects of the session and translations about the meaning for some topics.

The recorded sessions were transcribed. The process of this was specific to the language, spoken English or NZSL. The spoken English audio files were put into AI transcription software, (Otter.ai, 2024) for initial transcription. The transcription was then reviewed against the recording in full by the second author and amended for accuracy as needed. All participants who used spoken English (n = 6) were sent their transcripts for checking. One participant responded and adjustments were made to their transcript, according to feedback. The NZSL sessions were viewed, then translated and transcribed in English by the second author. Following this, a qualified NZSL interpreter reviewed a minimum of 10% of each session which was the most challenging to translate. There were few corrections to make. These were completed in consultation with the second author. The interpreter signed a confidentiality agreement, and participants were made aware of who the interpreter was, as is important in a small community of NZSL users. An honorarium was provided to the interpreter for his services. At the end of this process, some sections of some recordings remained unclear in their translation. All participants who used NZSL (n = 7) were asked to clarify sections of their transcripts from the recordings, and review the transcript overall, for accuracy. All participants responded and clarified the ambiguities. After being finalised, all transcripts (now in English) were entered into NVivo (QSR International, 2022) for coding.

## Data analysis

In alignment with the transformative research paradigm (Mertens, 2008), reflexive thematic analysis (Braun & Clarke, 2019) was used to analyse data from the focus groups and interviews, following an iterative, broadly non-linear process. The second author led this process. She immersed herself in the data by reviewing all video and audio recordings, as well as transcripts, several times before coding. She kept a reflexive journal throughout, capturing evolving thoughts, connections, and potential codes. She generated initial codes inductively, grouping data points that shared similar meanings or concepts. These codes were then reviewed and refined, occasionally merging several into one, or splitting one broad code into more specific and clearly defined codes in consultation with the first and third authors. Codes were then organised into potential themes by examining their alignment and shared narrative by the second author. Braun and Clarke’s (2019) definition (where a theme represents a central idea or shared meaning) guided this phase. A thematic map was created to clarify how these themes were related, and some were adapted or merged. Ultimately, three subthemes were developed, although reflection revealed a missing overarching theme. The main theme was identified through further analysis and reflective discussions between all authors. Subthemes were systematically reviewed, named, and documented, forming a coherent narrative of the findings in relation to the research questions by the second author. Final feedback on sense making regarding clarity and sufficiency of coding was provided by the first and third authors (Braun & Clarke, 2019).

In alignment with the principle of culturally appropriate data analysis (Smith, 2015), a reflexive conversation was held between the second author and a Māori cultural advisor during the analysis process, to review the thematic map, however this was not done with a Deaf cultural advisor (Kenway & Riley, 2021). Rather, an initial report, featuring a presentation and accompanying NZSL video, was shared with participants and they were invited to provide feedback on the completed analysis. Three offered general comments and encouragement, while one engaged in a more detailed discussion, suggesting adjustments around “relationship before task.” This participant described professionals adept at building rapport while completing tasks, prompting refinements to the relevant sections. The same participant reviewed their quotes to ensure clarity, making minor changes to ensure clarity. Although all participants could discuss their quotes, only one took the opportunity. After these revisions, the analysis was complete. ​.

In alignment with the Deaf AcCES checklist and kaupapa Māori research (Kenway & Riley, 2021; Smith, 2015), participants were consulted on how they would like the results to be disseminated. As a result, a Zoom meeting was held using NZSL with the Deaf community, and a one-page research summary and accompanying summary of the research as a video in NZSL was disseminated to the d/Deaf community, in addition to typical academic avenues, including two international conferences, and the current paper.

## Results

### Main theme: ensuring deaf children are thriving and flourishing

**Figure 1:**
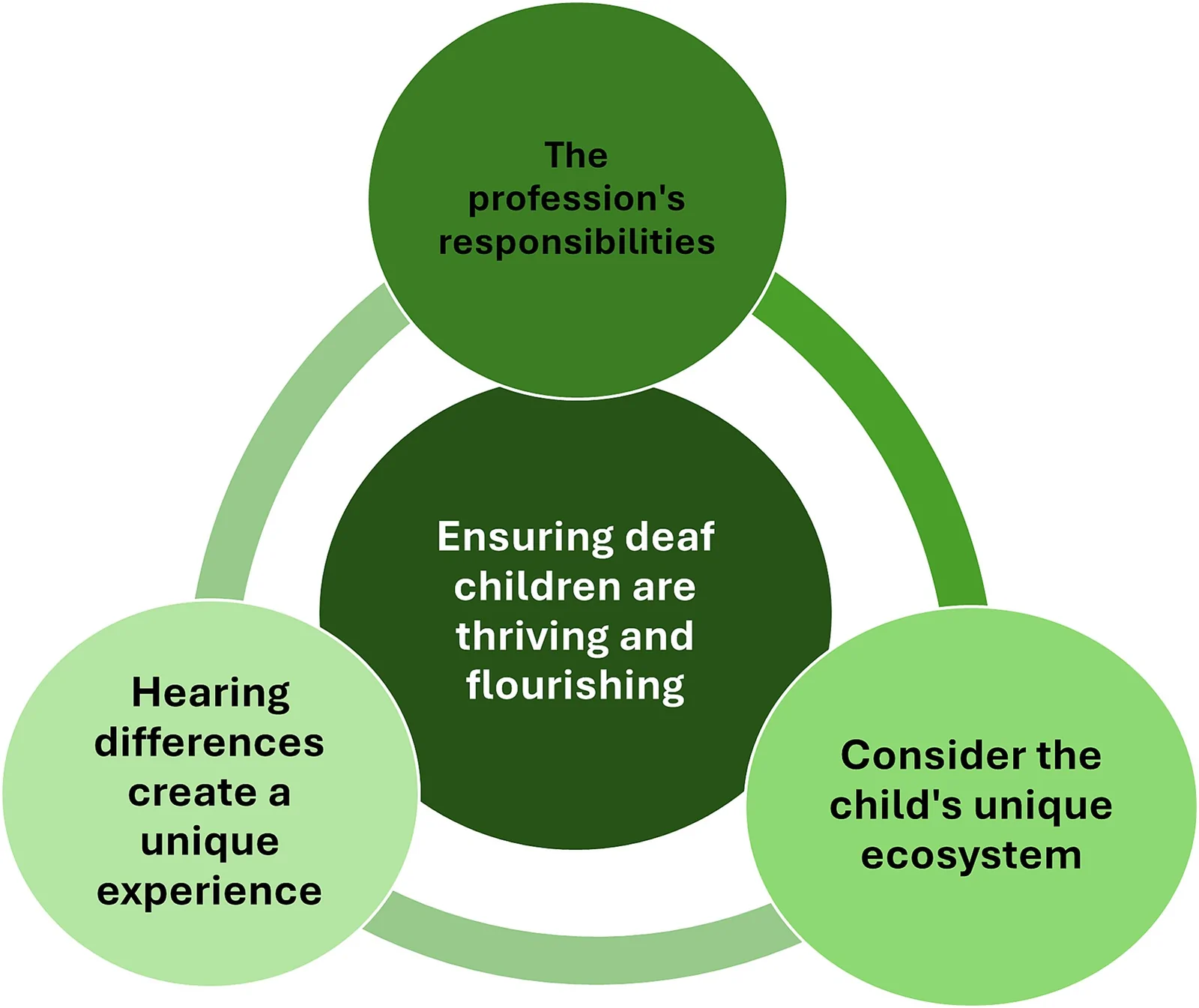
Theme and subthemes.

All participants emphasized the importance of supportive experiences that foster development and well-being for deaf children, whether in SLT or other contexts. This theme broadly answered the third research question (how should SLTs support deaf children?) by recommending a holistic approach that considers each child’s environment and relationships. Participants were aware of the influential role professionals can play, as James signed that support “needs to start at a young age to become a foundation that a child can grow from. Like sowing seeds that then grow into plants, you have an important job to make sure that the right seed is planted”. For example, Evelyn recalled her SLT “gave me strategies to become independent and talk to people with confidence.” Will noted how his SLT “started off my interest in music as well… I sort of dismissed it as not really for a deaf person.” Family influence was important too, for instance, Ngaroma underscored her grandmother’s positive influence: “Instead of making things a big deal, she would just put me where I could best hear and flourish.” Some participants shared aspirations for deaf children, such as James wanting “deaf kids growing up in happy environments… accepting of themselves.” Mary stressed the importance of early language access: “They need to be given sign language straight away, day one.” The following subthemes detail specific elements that enable deaf children to thrive. These were conceptualised as interconnected sub-themes supporting the main theme, as shown in Fig. 1. The subthemes, codes and subcodes are shown in Table 3.

**Table 3:** Subtheme coding tree.

Subthemes	Codes	Subcodes
Hearing differences create a unique experience	Access to language, identity and culture	Confidence is important
		Seeing myself; sharing lived experience
	Navigating towards successful communication	Lip reading
Consider the child’s unique ecosystem	Environment	
	Responsive people as part of the ecosystem	
	Each child and ecosystem will be different	
The profession’s responsibilities	Relationship and task	Balance power; provide autonomy
		SLTs need to be transparent and honest
	Diverse and culturally responsive workforce	

### Subtheme one: hearing differences create a unique experience

Participants emphasized that deaf individuals have distinctive lived experiences compared to those who develop with typical hearing. This subtheme highlights important considerations for SLTs working with deaf children.

Participants spoke of *access to language, identity and culture*. Participants insisted that both signed and spoken languages should be provided from the start. Mary signed, “I think NZSL is important for all deaf children. It doesn’t matter if they have cochlear implants or whatever. If they have acquired NZSL, they can also use their hearing to make connections between the languages” and “don’t make people choose. Just give all the languages”. Ngaroma also discussed the importance of the Māori language for Māori deaf children, emphasizing how cultural knowledge, identity, and heritage intertwine. However, she felt these needs were often overlooked. Reflecting on pūrākau (Māori stories) told exclusively in Māori, Ngaroma noted, “That’s what’s really sad for me: they’ll never hear those… mainly because they’re all in reo, but also because they don’t have the educators or the resources to access those pūrākau of Māori, Māori doing great things, but they might not have had all their senses”.

Participants also conveyed how *confidence is important*. They noted Deaf children need a sense of identity and confidence, but this is hard to build, growing up as a deaf person in a majority hearing world. Several participants recalled times when SLTs gave them reassurance about their spoken communication skills, only for them to encounter real-world challenges. James described this as “false hope,” while Rachel recounted a SLT who said, “‘You’ll be fine in the world, get a job,’” but real-life communication proved far tougher. Participants also believed *seeing myself; sharing lived experience* was important. Participants shared how their lived experiences could encourage families. Evelyn noted, “When I’ve talked to the parents of one of the [deaf] children at my work… they’ve appreciated hearing it from my own perspective.” Others wanted to see more successful deaf role models. Will expressed hope for “somebody who’s unashamedly deaf… just absolutely killing it,” highlighting the value of successful Deaf role models.

The code of *navigating towards successful communication* was developed. Spoken-language users reported having to balance social acceptance with advocating for accommodations, such as removing face masks during the COVID pandemic. Will explained that masks made him feel “like walking with one leg,” since lip reading was his default strategy, however asking people to remove their face masks felt socially awkward. Will also described the feeling of being “close enough to pass, but not quite perfect enough to actually be involved.” NZSL users faced parallel challenges, sometimes relying on family members to interpret or resorting to writing when communicating with hearing people. Some encountered wary reactions from hearing people unaccustomed to different communication methods. Assefa recalled, “I go into a shop, ask them to write it down, and they get freaked out and call the manager.” Evelyn praised her SLT for teaching pragmatic conversation skills including how to respond to people, which enhanced her confidence. Evelyn said she appreciated the SLT “explaining to me that there are people out there who don’t understand and they won’t know until somebody tells them. So… accept that that’s just the fact of life that I will always be explaining to people…and showing them there’s nothing wrong with me.”

Across both groups, *lip reading* was also emphasised. Participants stressed the importance of visual access to a speaker’s mouth or face. Chloe, an NZSL user, explained, “I would watch lip patterns to connect words,” while Mary called masks a complete barrier: “I can’t hear anything; I need to see the mouth.” Several participants criticized practices for teaching oral communication that withheld visual speech cues, such as covering the mouth or sitting behind the child. Chloe signed, “The thing I hated most was covering the mouth… I hated that!” Allen emphasized linking visible lip patterns with sound production: “It’s so much better to see the whole face.”

Overall, participants wanted SLTs to provide signed and spoken languages early, acknowledge cultural contexts (including cultures associated with ethnicity), and to focus on confidence-building to help mitigate the extra challenges deaf people face in daily communication. They noted recognizing each individual’s distinct experience and context enables SLTs to empower deaf children and their families more effectively.

### Subtheme two: consider the child’s unique ecosystem

This subtheme focuses on viewing the deaf child within their broader ecosystem, emphasizing how unique contexts, supportive relationships, and family networks are essential. Firstly, participants noted the importance of the *environment*. Participants stressed that SLTs should observe children’s abilities in everyday settings, not solely in clinical or school-based contexts. Assefa proposed, “It would be a good strategy for the SLT to be flexible and see the child in their natural environment… and fit into the child’s world.” Similarly, Ngaroma suggested that “understanding the world the child comes from and what communication is helpful” helps SLTs support deaf children to succeed in authentic interactions.

Secondly *responsive people as a part of the ecosystem* were emphasised. Many participants highlighted that a deaf child’s well-being depends on the ecosystem of support from family, friends, and educators. Evelyn commented, “It’s not just the child taking the journey on its own.” Several advocated for broader assistance, with Ngaroma explaining that her hearing difference affected her entire whānau (family): “It’s not just my hearing loss, it’s like that whakataukī, ehara taku toa, i te toa takitahi, and so something that I win is not my win alone, it’s the win of the collective. Same thing with disability, I think it’s not my, it’s not just my hearing loss. It’s been a real, it’s been a real burden for my family”. Mary, a Deaf parent, wanted more guidance for hearing relatives on how to communicate with her son: “He’s deaf, so you need to remember to make eye contact.” James added, “There needs to be strong support for children from their parents and the school, working together.”

Participants shared examples of social environments that enabled them to flourish. Ngaroma’s grandmother proactively adjusted Māori cultural protocols, allowing Ngaroma to sit at the front during pōhiri (welcome ceremony), including learning to do the karanga (welcome call), so she could fully participate. Ngaroma explained “I guess instead of making it a big deal… she would just put me where she knew that I would best hear and flourish… She never made it my problem”. Reflecting on this, Ngaroma wondered, “If it’s hard for me, and I’ve had a great experience, imagine what it’s like for everyone else?” Others spoke about individuals who actively supported their communication. Evelyn credited her mother for adapting strategies, while Will praised friends who filled him in whenever he missed details: “They’re easier people to hang out with.”

Finally, they noted *each child and ecosystem will be different*. Participants warned against oversimplifying deaf children’s needs, noting the wide diversity in the deaf community. Allen insisted that SLTs must “adjust to that individual,” and Ngaroma emphasized that success in communication “is really individualized to the child and the family.”

Overall, Subtheme Two underscores how SLTs can better support deaf children by considering each child’s unique environment, fostering strong support networks, and avoiding a one-size-fits-all approach.

### Subtheme three: the profession’s responsibilities

This subtheme outlines participants thoughts on the profession’s responsibilities. Participants commented on *relationship and task*. All participants underscored how a SLT’s approach and demeanour can shape a child’s therapy experience. Many recalled how SLTs made them feel as children, rather than the techniques they used. Will noted, “You don’t remember stuff inside particular lessons; [you] remember the teachers and how they treated you...” Hannah added that she connected more quickly with a SLT who signed fluently, and Evelyn praised a SLT whose “demeanour…made you always feel good in her presence.” In contrast, some described negative interactions where professionals seemed dismissive. Hannah, a Deaf parent, felt sidelined by her deaf child’s SLT, despite her own expertise on lip reading. Chloe recalled encountering SLTs who exaggerated their mouth movements when addressing her, leaving her feeling patronized.

Participants noted it was important to *balance power; provide autonomy*. Assefa noted some SLTs “don’t ask me for my opinion about anything,” and Chloe worried about newcomer SLTs in her workplace who might “take over” rather than cooperate with Deaf colleagues. Some participants, when they were deaf children, felt forced into therapy without being consulted. Assefa recalled, “I felt forced into it… no one gave me the choice.” James described being forced to listen and use spoken language: “I was dragged into their world, what choice did I have, I couldn’t argue with them, I was a child”. Others expressed frustration by dawdling on route to sessions to reduce the time spent there. Physical boundaries emerged as well: some disliked having their hands placed on a SLT’s throat to feel vibrations. Will, however, described a positive experience where his SLT respected his autonomy and presented clear options.

Participants also communicated that *SLTs need to be honest and transparent*. Participants recounted how a SLT’s words can have a significant impact on a deaf person’s self-perception and confidence particularly about their chances of integrating into the hearing world. James termed some interactions with his SLT as giving “false hope,” and Hannah wished for more specific guidance on how to speak and the effort she’d put in, instead of “you’re great…off you go.”

Finally, participants commented on the need for a “**diverse and culturally responsive workforce**.” Participants noted the homogeneity of the SLT profession, typically young hearing white women, and argued that greater diversity in gender, ethnicity, and hearing status could foster deeper connections and more culturally competent care. Rachel pointed out the benefits of having male SLTs for boys or SLTs from various ethnicities to match the families’ backgrounds. Ngaroma highlighted that Māori deaf children, already at risk of losing cultural connections, need culturally affirming professionals. For participants using NZSL, having the “right attitude” mattered greatly here. Chloe emphasized openness to Deaf culture and sign language, and Rachel stressed a “positive attitude” without judgment. Several participants advocated for more collaboration between SLTs and Deaf adults, suggesting that Deaf role models or interpreters in therapy sessions could enhance outcomes. Participants observed that deaf children vary widely in backgrounds and needs, so professionals must remain flexible.

Overall, Subtheme Three highlights the profession’s ethical responsibility to foster supportive relationships that empower deaf children and families. This involves acknowledging power dynamics, respecting autonomy, and being honest about communication goals and challenges. By embracing diversity and cultural responsiveness, SLTs can better serve a heterogeneous deaf population. As participants insisted, “the right attitude” and willingness to learn from Deaf individuals are key to truly effective partnerships.

## Discussion

This qualitative study reports on the experiences and perspectives of 11 deaf adults from New Zealand on SLT and how the profession should support deaf children. The study used the socio-linguistic, cultural and ecological systems models of Deafness as its framing (Cue, 2020; Ladd & Lane, 2013). This study drew on a transformative research paradigm; principles of Kaupapa Māori research; the Deaf AcCES checklist and consultation with community stakeholders and Ngāi Tahu as local iwi (Kenway & Riley, 2021; Mertens, 2008; Smith, 2015). Data was generated in aged-based focus groups and semi-structured interviews via Zoom, in either NZSL or English, depending on the participants preference. Audio and video recordings were used for transcription. NZSL was translated into English. Reflexive thematic analysis was used to analyse the data (Braun & Clarke, 2019). One main theme and three subthemes were generated, which are discussed below with reference to theoretical models of deafness, the literature and the New Zealand context. Clinical implications are discussed throughout.

### Main theme: ensuring deaf children are thriving and flourishing

Participants collectively emphasised that SLTs must support deaf children to thrive as whole people. Feedback suggested some SLTs focus too narrowly on listening and spoken language, overlooking identity development, confidence, communication skills, and self-acceptance. Findings therefore support a holistic health model, rather than a medical one targeting “impairment” (Haegele & Hodge, 2016). These experiences are notable, given the NZSTA’s scope of practice actually endorsed use of the ICF model (WHO, 2001), child- and family-centred practice (Allen & Petr, 1996), and cultural competence (NZSTA, 2012). Although some participants’ interactions with therapists occurred before this scope of practice was published, within participant answers, there was no clear impact of a difference across time. Those who currently had children who were deaf or interacted with therapists as colleagues more recently made similar comments to those who received therapy as a child. However, there was a trend towards participants who communicated in NZSL as compared with spoken English reporting more negative experiences with SLTs. Tensions remain between the themes identified here and the profession’s endorsed models, suggesting ableism (St. Pierre & St. Pierre, 2018) continues to constrain their application with deaf children. These issues are examined further in the subthemes below.

### Subtheme one: hearing differences create a unique experience

#### Access to language, identity, and culture

Participants emphasised the importance of access to Deaf culture, signed language*,* and the Māori culture and language, aligning with the sociolinguistic and cultural models of Deafness (Ladd & Lane, 2013). They strongly supported providing deaf children with both signed and spoken languages from birth, enabling families to use the mode that bvest suits the child. This aligns with the World Federation of the Deaf’s declaration of deaf children’s right to sign language (2016) and evidence that signed language access prevents language deprivation (Hall et al., 2019; Sanzo, 2022; Spellun et al., 2022). Deaf adults and experts in deaf education in previous studies likewise valued inclusion of sign language in therapy (Cripps et al., 2016; Greene et al., 2023; Sankey, 2019; Secora et al., 2025; Veyvoda et al., 2019).

However, the principle of “family choice,” central to family-centred care (Allen & Petr, 1996; Ministry of Education, 2021; NZSTA, 2012) may lead to conflict with deaf adults’ views on what is best for deaf children. In New Zealand, families choose whether their deaf child will have access to NZSL or not. This may create tension for SLTs navigating between deaf adults’ perspectives on the importance of access to d/Deaf identify, sign language and Deaf culture, and families favouring oralist approaches. Over 90% of deaf children are born to hearing parents (Mitchell & Karchmer, 2004). For families choosing NZSL, support to learn it is limited, particularly in rural areas (Ministry of Education & Fitzgerald & Associates, 2019). Parents of deaf children weigh the communication advice of SLTs highly (Jones & Roberts, 2024). At a minimum, families need open, informed discussions about communication options, including deaf adults’ perspectives, research evidence, and the availability of supports (Jones & Roberts, 2024; NZSTA, 2012, 2020; Terry & Rance, 2023). Aligning with participants’ recommendations would also require SLTs to champion Deaf culture and to be competent in NZSL.

Ngaroma also commented on the importance of Māori deaf children having access to their culture and the Māori language. This is in alignment with Crisp (2010) who reported in their master’s thesis that most Māori whānau (families) wanted their deaf child to learn the Māori language and access Māori culture. Smiler and McKee (2006) also described how Māori deaf experienced a lack of cultural knowledge, reliance on their hearing family to communicate, and difficulty assuming expected Māori roles. They also noted the tension of moving between Māori and Deaf worlds, with identity development depending on access to peers and role models.

#### Seeing myself; sharing lived experience

Identity development through role models and peer connections was seen as vital in the current study and is in alignment with the sociolinguistic and cultural models of Deafness (Ladd & Lane, 2013). The National Deaf Children’s Society (2011) also found that young deaf people sought identity support, peer connection, and reduced isolation. Terry and Rance (2023) identified identity support as a key theme in their scoping review of systems which support families of deaf children.

Two unpublished local studies also support this finding, including for Māori families (Crisp, 2010; Smiler, 2014). Conversely, Greene et al. (2023) observed that some of their deaf participants felt judged when compared with peers, with their worth linked to speech ability. Collectively, this evidence highlights the importance of peer and role model access, though comparisons and deficit framings are to be avoided.

SLTs could help facilitate this by connecting families with Deaf mentors and community events, supporting deaf peer groups, and incorporating Deaf role models and stories into therapy sessions. They could also collaborate with teachers of the deaf to create environments that validate multiple communication modes and identities, fostering belonging and positive self-concept among deaf children. Similar supports could be provided to support deaf children to access their ethnic heritages, including Māori cultural identity, in collaboration with local communities.

#### Navigating towards successful communication

Participants described challenges across contexts, regardless of language used. They wanted support for functional everyday communication outside clinic settings. Greene et al. (2023) similarly reported participants valued SLT support for independence in real-world communication, while Sankey (2019) highlighted frustration with lack of support for group interactions. Research with mainstreamed deaf teenagers showed recommended supports for pragmatics (Zaidman-Zait & Most, 2020). These findings align with NZSTA (2020), the ICF (WHO, 2001) which prompt activity, participation, and environment-focused goals. He Pikorua (Ministry of Education, 2021), the practice framework for SLTs working for the Ministry of Education Learning Support Service, also aligns with these recommendations. SLTs should therefore promote communication success in diverse contexts, and not limit their practice to auditory-verbal and or sign language skills in pull-out settings.

Finally, participants stressed the need for visual access to the face and disapproved of covering the mouth as a therapeutic practice. They may have been referring to AVT methods that discourage lip reading. Evidence from mask use during the COVID pandemic shows visual cues (alongside auditory cues) support speech perception (Badh & Knowles, 2023). While AVT overall has positive outcomes for language and speech perception (Binos et al., 2021; Noel et al., 2023), it is unclear whether covering the mouth is necessary to facilitate these outcomes.

### Subtheme two: consider the child’s unique ecosystem

This subtheme highlights participants’ emphasis on considering all aspects of a child’s life and environments, and the dynamic ways in which children interact with those contexts. Participants stressed the importance of seeing the child within their *environment* and the value of *responsive people as part of the ecosystem* around the child, including providing families with tools to support successful communication. This aligns with the Deaf Ecological Systems Model (Cue, 2020), which explicitly centres the deaf person as a system in their own right, while also emphasising family, cultural, and institutional layers. He Pikorua is also a child- and family-centred ecological model, focused on inclusion through collaboration with everyday communication partners (Ministry of Education, 2021). The ICF model (WHO, 2001) prompts therapy goals to change the physical and social environment around the client. Terry and Rance (2023) and Nightingale et al. (2025) underscored the importance of enabling deaf children’s participation in everyday life settings through social and environmental supports.

To align with these findings, SLTs may adopt ecological assessment approaches that encompass family, school, and community contexts, ensuring that intervention goals are meaningful across settings. They can collaborate with teachers, interpreters, and Deaf mentors to strengthen the communicative environment and promote inclusion. Supporting families to embed communication opportunities within everyday routines and advocating for system-level changes (such as enhanced NZSL access and culturally sustaining practices within schools) further reinforces this ecological perspective. In doing so, SLTs move beyond individual intervention to facilitate interconnected, communication-rich ecosystems that promote participation and belonging for deaf children.

Finally, participants recognised the need for family-centred approaches that respect diversity, acknowledging that *each child and family ecosystem is different.* This is in keeping with best practice in early intervention, which prioritises family-centred service delivery grounded in families’ choices, strengths, needs, and relationships with professionals (Allen & Petr, 1996; NZSTA, 2020).

A practice model developed specifically for deaf children in the New Zealand context, including adhering to obligations under the te Tiriti o Waitangi, including the recommendations above, would be a highly useful addition to the clinician’s toolkit.

### Subtheme three: the profession’s responsibilities

#### Relationship and task

Bright and Reeves (2022) and Hansen et al. (2024) emphasised that accommodating communication needs is central to therapeutic relationships. They found clients actively evaluated SLTs and other professionals, judging behaviours and motivations. Participants in this study reported doing the same, noting that perceptions of therapists directly affected the relationship. Bright and Reeves (2022) also highlighted the importance of seeing each other as people and balancing power. Several participants described power dynamics: some felt forced into therapy methods, while others valued the autonomy therapists offered. Hansen et al. (2024), Bright and Reeves (2022), and Sankey (2019) noted patients appreciated therapists who adjusted communication, including use of sign language. Conversely, Hansen et al. (2024) and Bright and Reeves (2022) found disregard or patronisation caused disengagement, which participants in this study also reported. SLTs must therefore reflect on how they communicate, share power, and foster respectful, trusting relationships with deaf children.

#### Diverse and culturally responsive workforce

This study reports deaf adults’ concerns about workforce diversity. As expected from prior research (Newbury et al., 2020), participants raised the lack of SLTs’ sign language proficiency, but they also were critical of limited gender and ethnic diversity. Figure New Zealand Trust data from the 2018 census (Figure NZ Trust, 2024) showed only 2.9% of SLTs were male. Brewer and Andrews (2016) found the profession also failed to reflect New Zealand’s ethnic composition, with Māori, Pacific, and Asian peoples underrepresented. Comparable concerns have been noted in the UK and Australia (Begum, 2024; Boyd & Hewlett, 2001; Byrne, 2015). Acknowledging this, NZSTA’s 2020–2025 strategic plan committed to “providing leadership in building a diverse workforce with a wide range of skills for the future” (NZSTA, 2022, p. 3).

### Strengths and limitations

A strength of this research is the variety of participant experiences. A broad age range reflected different access to technology and education, though analysis showed no clear rationale for separating data by age group. Additional diversity came from variations in hearing levels and language use (spoken, written, and signed). However, only one Māori and one Indian participant were included, with the rest New Zealand European, and no Pacific peoples represented, so further research to show diversity of cultural views is recommended. This study drew on a transformative paradigm (Mertens, 2008), the Deaf AcCES checklist (Kenway & Riley, 2021) and kaupapa Māori research (Smith, 2015), with input in design from deaf and Māori community stakeholders. High quality data collection, transcription and analysis practices were utilised, with several opportunities for participants to shape the results of the study. Funds did not allow a professional interpreter to complete the full interpretation of the NZSL data, however adequate measures were taken to ensure the quality of the interpretation. A Deaf cultural advisor was not appointed, raising the possibility of researcher assumptions or biases about Deaf people. Participants were self-selected and may have been motivated by negative experiences of SLT. Some accounts described events from many years ago and none were verified. The data in this study have not been triangulated with other sources. A choice of interview or focus group format for data collection was given in response to participant request (Kenway & Riley, 2021), however these different methods of data collection can shape the type of data collected. For example, the focus group data may have yielded more information about shared perspectives, whereas the interview data may have shown only individual experiences or perceptions. However, participants were in high agreement with each other and with similar literature. The perspectives of SLTs themselves were not included and may differ from those of the participants.

### Future research

As noted, a local practice framework for working with deaf children could support SLTs to deliver Deaf culture–affirming practice. Such a framework should be evaluated by deaf clients, their families, and the SLTs applying it. To our knowledge, this is the only study to ask indigenous deaf people about their experiences of SLT. Similar research in countries with colonial histories (e.g., USA, Australia, Canada) would provide evidence to inform culturally appropriate services and enable comparisons across indigenous communities. Finally, existing studies, including this one, have relied on adult participants. Research involving children and teenagers currently receiving SLT would add valuable insight, capturing perspectives and nuances that may be lost when reported retrospectively in adulthood.

## Conclusion

This study shows these deaf adults emphasised that flourishing for deaf children extends beyond listening and spoken language to include identity, confidence, access to both signed and spoken languages, cultural belonging, and participation in everyday communication. Although these priorities align with frameworks such as the ICF (WHO, 2001) and NZSTA’s scope of practice and code of ethics (2012; 2020), tensions emerged between endorsed models and reported clinical experiences, suggesting ableism continues to shape service delivery, possibly due to family preferences. Findings highlight the need for SLTs to provide balanced information to families on language access options, champion Deaf and other cultures, ensure children connect with deaf peers and role models, reflect on therapeutic relationships, use family-centred ecological approaches and improve diversity in the workforce. A locally grounded framework integrating ecological, cultural, and holistic perspectives could support bridging the gap between policy and practice. Future research involving underrepresented ethnic communities and deaf children themselves is needed to ensure services meet the diverse needs of deaf children.
